# Risk Factors for Fatal and Nonfatal Road Crashes in Iran

**DOI:** 10.5812/ircmj.10016

**Published:** 2014-08-05

**Authors:** Mohammadreza Mehmandar, Hamid Soori, Mosa Amiri, Reza Norouzirad, Mehdi Khabzkhoob

**Affiliations:** 1 NAJA Research Center of Traffic Police of Iran, Tehran, IR Iran; 2 Safety Promotion and Injury Prevention Research Center, Shahid Beheshti University, Tehran, IR Iran; 3 Biochemistry Department, Faculty of Medicine, Dezful University of Medical Sciences, Dezful, IR Iran; 4 Department of Epidemiology, Faculty of Health, Shahid Beheshti University of Medical Sciences, Tehran, IR Iran

**Keywords:** Cross-Sectional Study, Iran, Risk Factors

## Abstract

**Background:**

Road traffic injuries are among the leading causes of death in the world and Iran.

**Objectives:**

The aim of this study was to assess the role of age, sex, education, and time of accident on human casualties and mortalities of road crashes in Iran.

**Materials and Methods:**

This study was based on data gathered by Iranian Police Department from the records of road crashes from April 4, 2008 through April 4, 2009. Road crashes are categorized into three types: with no human casualties, with injuries, and with human mortalities.

**Results:**

The largest rate of human causalities was observed in people aged between 25 to 34 years (P < 0.001). Illiterate people had 81% smaller odds of causality in road crashes (P < 0.001) in comparison with those with a kind of academic education. Overall, 73.4% of crashes had happened during the last ten days of a month were with human casualties (P < 0.001) and human casualties rate was slightly higher in crashes happened between 1 AM to 5 AM Fatality rate was slightly higher in the females (OR = 2.6, P = 0.068). The smallest odds of fatality were found in the people aged between 18 to 24 years and the highest odds were seen in people ≥ 55 years of age (P < 0.001). In people with a university education, 61.9% of crashes were with fatality (P = 0.026). In addition, 82.8% of crashes during winter, 60.2% of crashes during autumn, and 35.8% of crashes during summer were with mortalities. Overall, 78.3% of crashes with human casualties that had happened during 1 AM to 5 AM led to mortalities. There was also a significant association between injury and its intensity with fastening seatbelts.

**Conclusions:**

Older age, university degrees, female sex, wintertime, and the time of accident seem to be the most important risk factors in road crashes that lead to fatalities in Iran. Drivers in Iran should be informed and trained regarding these risk factors, which have direct effect on casualties and mortalities in road crashes.

## 1. Background

Road traffic injuries are among the leading causes of death in the world and have direct impact on the well-being of the societies (1). According to the World Health Organization (WHO), road crashes are the ninth most frequent cause of death, especially in countries with low or average income (2). By detecting the risk factors in each country, we can use preventing measures to control the effects of the risk factors in road crashes and finally, reduce the rate of casualties. Sex, age, and education are important factors in road crashes in various societies (3-9). Other human factors such as driving under influence of drugs, (10, 11) low vision acuity of driver, and even poor hearing (6) have been identified as effective factors in road crashes. Several reports indicate an approximate number of 17 million vehicles in Iran, which is the largest number in East Mediterranean region (12). Human mortality rate by road crashes are 35.8 in 100000, which makes Iran one of the top countries in east Mediterranean region in this regard (12). It has been estimated that traffic crashes will double by 2030 (1); therefore, our country would be affected dramatically with effects of road crashes in future. According to the report by Soori et al. (13), transport accidents in Iran were the leading cause of fatal and nonfatal injuries regardless of the accidents types.

## 2. Objectives

Studying the risk factors that are associated with crashes and their fatalities helps to find measures for controlling them to reduce their fatalities. A vast number of studies have been conducted to evaluate such factors in Iran but a lot of them have been performed in restricted areas of country. Therefore, there is not a detected risk factors pattern on the road crashes in Iran. In this study, we tried to evaluate the risk factors for casualties and mortalities of car crashes in the whole areas of Iran.

## 3. Materials and Methods

**Figure 1.**
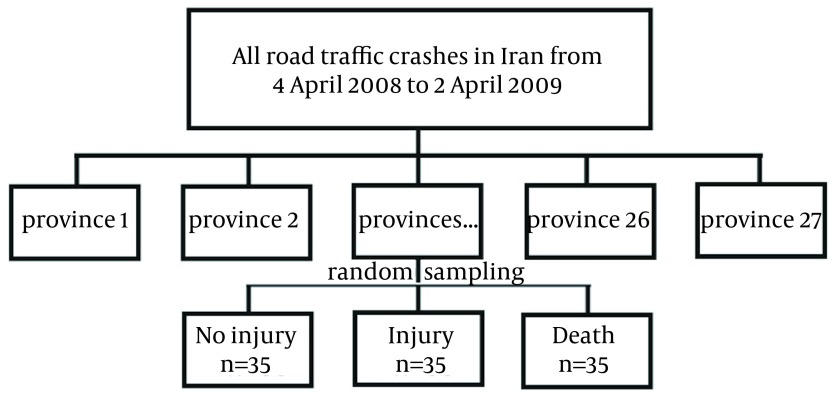
Sampling Process Flowchart

Our study was a cross-sectional analytic study based on the data recorded by Iranian Police Department from all of road crashes in 30 provinces in the country from April 4, 2008 to April 4, 2009. Target population in this study were the involved people in the road crashes within this period. The process of selecting samples in our study is shown in Figure 1. We chose 105 samples of road crashes from each province. A total of 35 samples for each type of crashes were selected. Road crashes were classified into three types: crashes with no human casualties, crashes with injuries, and crashes with human mortalities.

Data for each sample of this study was gathered based on a standard accident report that was filled by a police officer who was present at the site of accident. The recorded parameters in the accident report helped us to compare the road crash samples with each other. Police officers wrote down these parameters in the reports at the time of accident. These parameters included time of crash, place of the crash, type of plaintiff vehicle, type of involved vehicles, crash type, road and weather condition, the main reason of crash, road defects, visibility at the time of crash, effects of human errors on the crash, status of fastening seatbelts, and demographic characteristic of the involved people in the crash. Each accident report with two or more uncompleted parameters were excluded from the study.

Accidents in intercity roads and occurrence of the accidents from April 4, 2008 through April 4, 2009 were the inclusion criteria. After selecting the samples, the cases with undetermined outcomes and those incompletely recorded dependent variables were excluded. Data from three provinces were incomplete and therefore, their information was excluded, which decreased the total number of the study samples to 2579 from 27 provinces. Out of 27 provinces, 945 samples with each type of accident were selected. A total of 2835 accidents were selected through systematic stratified sampling. After implementing the eligibility criteria, due to incomplete records, 892 accidents out of 945 accidents without human injury (94.4%), 882 accidents out of 945 accidents with human injury (93.3%), and 686 accidents of the 805 accidents resulting in human death (85.2%) were analyzed.

### 3.1. Statistical Analysis

We analyzed data in our study by STATA v. 8.0 (STATA Corporation, College Station, TX, USA). First, we evaluate the association between some of the parameters by Chi Square test. We performed data analysis in two steps to evaluate the effect of some parameters on crashes with causalities and crashes with mortalities. In the first step, we made a two-conditional parameter in which one condition was crashes with no injuries and the other condition was crashes with causalities or crashes with mortalities. In the second step, we evaluated the parameters that affected crashes with mortalities. We divided the main parameter according to two conditions: crashes with causalities and crashes with mortalities. For this part of our analysis, we used simple and multiple logistic regressions. To show the power of our study, we used odds ratio with 95%confidence interval (95% CI). Considered risk factors in our study were age, sex, education level, time of day and season of the year when crashes had happened, status of seatbelts fastened by the involved people in the crashes, and the day of the month when crash had occurred.

### 3.2. Ethical Considerations

This study was approved in the 116th meeting of Safety Promotion and Injury Prevention Research Center in April 2012. All the reports were performed anonymously for ethical considerations.

## 4. Results

Table 1 shows the characteristics of the involved people in the crashes in our study. This table shows that there was not any significant difference between sexes in crashes with causalities or fatalities. On the other hand, age had a significant difference in the various kinds of crashes. The largest fatality rate was seen in the age group of 25 to 34 years (29.4%). In addition, there was a significant difference between education levels of the involved people in the crashes. Illiterate people had the highest fatality and causality in the crashes while those with academic degrees had the lowest rate of the causality and mortality (P < 0.001). Another important risk factor in our study was fastened seat belts. In other words, 57% of the mortalities and 48.2% of causalities were seen in people who had not fastened their seatbelts at the time of accident. In the crashes with no injuries, 99% of involved people had fastened their seatbelts at the time of crash (P < 0.001). 

The season of the year when the crashes had occurred has a major role on the rate of fatalities in road crashes in Iran. The highest and the lowest fatality rates were seen in the wintertime and the summertime, respectively (P < 0.001). In this study, a high percentage of human causality and fatality had occurred from 20th through 30th day of the Persian calendar. Table 1 shows the time of the day when crashes had happened. Time has great effects on the causalities and mortalities of the crashes. The highest mortality rate was seen in the crashes that had happened between 1 AM to 5 AM while highest causality was seen in the crashes happened between 6 PM to midnight, the lowest causality rate was seen in the crashes happened between 6 AM to 11 AM (P < 0.001).

**Table 1.** The Distribution of Independent Variables According to Studied Groups ^a^

	No Injury	Injury	Death	P Value
**Gender**				0.170
Male	888 (34.7)	877 (34.3)	793 (31)	
Female	10 (37.0)	5 (18.5)	12 (44.4)	
**Age**				< 0.001
24	107 (17.5)	316 (51.7)	188 (30.8)	
25-34	99 (17.8)	221 (39.7)	237 (42.5)	
35-44	401 (54.2)	170 (23.0)	169 (22.8)	
45-54	231 (48.7)	115 (24.3)	128 (27.0)	
≥ 55	58 (29.0)	59 (29.5)	83 (41.5)	
**Education**				< 0.001
Illiterate	288 (25.9)	434 (39.1)	388 (35.0)	
not college	491 (38)	424 (32.8)	378 (29.2)	
College	119 (65.4)	24 (13.2)	39 (21.4)	
**Seat belt**				< 0.001
Used	858 (51.7)	457 (27.0)	346 (20.4)	
Not used	40 (4.3)	425 (47.6)	459 (51.5)	
**Season**				< 0.001
Spring	156 (22.3)	321 (45.9)	222 (31.8)	
Summer	214 (25.5)	402 (47.9)	224 (26.7)	
Autumn	262 (45.9)	123 (21.5)	186 (32.6)	
Winter	266 (56.0)	36 (7.6)	173 (36.4)	
**Day of month**				< 0.001
1-10	414 (45.2)	255 (27.8)	247 (27.0)	
11-20	250 (31.7)	290 (36.8)	248 (31.5)	
20-30	234 (26.6)	337 (38.3)	310 (35.2)	
**Time of day**				< 0.001
1-5	59 (35.8)	23 (13.9)	83 (50.3)	
6-11	334 (43.4)	210 (27.3)	226 (29.4)	
12-17	242 (30.5)	309 (38.9)	243 (30.6)	
18-24	263 (30.7)	340 (39.7)	253 (29.6)	

^a^Data are presented as No. (%).

### 4.1. Effect of the Risk Factors on the Rate of Injuries 

Table 2 shows risk factors in road crashes with human injuries based on independent variables. Our study analysis showed that there was a significant association between age and rate of injury, but sex had not shown such an effect. We found out that there was no significant difference between the odds of getting injured and age of involved people in the crash in the age groups of ≤ 24 years and between 25 and 34 years of age. On the other hand, people who aged ≥ 34 years had better odds of not getting injured. Education was the other variable that decreased the odds of getting injured (P < 0.001). Involved people who had fastened their seatbelts in the crashes had lower odds of getting injured (4.5%). On the other hand, people who had not fasten their seatbelts were more likely to get injured (52.4%) (P < 0.001). In our study, we detected that road crashes with human injuries had happened more likely in springtime than in wintertime or autumn. In addition, data showed that road crashes with human injuries were more likely to happen in the last ten days of each Persian month. Overall, 73.4% of involved people in this kind of crashes had some injuries but only 54.8% of people, who were involved during the first ten days of Persian month, had been injured (P < 0.001). Comparing seriousness of injuries between 1 AM to 5 AM with those between 6 AM to 11 AM, road crashes happened between 1 AM to 5 AM were more dangerous (P = 0.073).

**Table 2.** The Association Between Risk Factors and Injury by Simple Logistic Regression Model ^a,b^

	No injury	Injury	OR (95% CI)	P Value
**Gender**				0.801
Male	888 (34.7)	1670 (65.3)	1	
Female	10 (37.0)	17 (63.0)	0.9 (0.41-1.98)	
**Age**				
24	107 (17.5)	504 (82.5)	1	-
25-34	99 (17.8)	458 (82.2)	0.98 (0.73-1.33)	0.907
35-44	401 (54.2)	339 (45.8)	0.18 (0.14-0.23)	< 0.001
45-54	231 (48.7)	243 (51.3)	0.22 (0.17-0.29)	< 0.001
≥ 55	58 (29.0)	142 (71.0)	0.52 (0.36-0.75)	< 0.001
**Education**				
Illiterate	288 (25.9)	822 (74.1)	1	-
Not college	491 (38.0)	802 (62.0)	0.57 (0.48-0.68)	< 0.001
College	119 (65.4)	63 (34.6)	0.19 (0.13-0.26)	< 0.001
**Seatbelt Use**				< 0.001
Yes	858 (51.7)	803 (48.3)	1	
No	40 (4.3)	884 (95.7)	23.1 (16.9-32.9)	
**Season**				
Spring	156 (22.3)	543 (77.7)	1	-
Summer	214 (25.5)	626 (74.5)	0.84 (0.66-1.06)	0.149
Autumn	262 (45.9)	309 (54.1)	0.34 (0.27-0.43)	< 0.001
Winter	266 (56.0)	209 (44.0)	0.23 (0.18-0.29)	< 0.001
**Day of Month**				
1-10	414 (45.2)	502 (54.8)	1	-
11-20	250 (31.7)	538 (68.3)	1.77 (1.46-2.16)	< 0.001
20-30	234 (26.6)	647 (73.4)	2.28 (1.87-2.78)	< 0.001
**Time of Day**				
1-5	59 (35.8)	106 (64.2)	1	
6-11	334 (43.4)	436 (56.6)	0.73 (0.51-1.03)	0.073
12-17	242 (30.5)	552 (69.5)	1.27 (0.89-1.81)	0.184
18-24	263 (30.7)	593 (69.3)	1.26 (0.88-1.78)	0.203

^a^Abbreviations: OR, odds ratio; and CI, confidence interval.

^b^ Data are presented as No. (%).

### 4.2. Effect of Risk Factors on Mortalities

Table 3 shows distribution of the variables in our study in two separated groups: road crashes with human injuries and road crashes with human mortalities. We found out that sex had an important effect on the outcome of the road crashes, especially on mortality rate. The mortality rate of females was about 2.6 times as high as that of males. Our analysis showed that 70.6% and 47.5% of involved females and males, respectively, were deceased (P = 0.068). Data analysis for age and mortality rate showed that people who were < 24 years old had lower mortality rate than those who were ≥ 55 years old (P < 0.001). Mortality rate of people with an academic degree was 61.9%, which was significantly higher than that of the illiterate people (P = 0.025). Analysis of data about the effect of the season showed that the mortality rate was 82.8% in wintertime, 60.2% in autumn, and in 35.8% in summertime, which is the lowest rate. There was no significant association between the mortality rate and the day of the month when crashes had occurred. Our analysis showed that the road crashes that had happened between 1 AM to 5 AM had more serious outcome and higher mortality rates (78.3%). Finally, our analysis showed a significant difference in mortality rate between those who had fastened their seatbelt with those who had not (41.3% and 51.9%, respectively; P < 0.001).

**Table 3.** The Association Between Risk Factors and Fatality by Simple Logistic Regression Model^a,b^

	Injury	Death	OR (95%CI)	P Value
**Gender**				0.068
Male	877 (52.5)	793 (47.5)	1	
Female	5 (29.4)	12 (70.6)	2.65 (0.93-7.57)	
**Age**				
≤ 24	316 (62.7)	188 (37.3)	1	
25-34	221 (48.3)	237 (51.7)	1.8 (1.39-2.33)	< 0.001
35-44	170 (50.1)	169 (49.9)	1.67 (1.26-2.21)	< 0.001
45-54	115 (47.3)	128 (52.7)	1.87 (1.37-2.55)	< 0.001
≥ 55	59 (41.5)	83 (58.5)	2.36 (1.62-3.46)	< 0.001
**Education**				
Illiterate	434 (52.8)	388 (47.2)	1	-
Not College	424 (52.9)	378 (47.1)	0.99 (0.82-1.21)	0.978
College	24 (38.1)	39 (61.9)	1.82 (1.07-3.08)	0.026
**Seatbelt Use**				< 0.001
Yes	457 (56.9)	346 (43.1)	1	
No	425 (48.1)	459 (51.9)	1.43 (1.18-1.73)	
**Season**				
Spring	321 (59.1)	222 (40.9)	-	-
Summer	402 (64.2)	224 (35.8)	0.81 (0.64-1.02)	0.073
Autumn	123 (39.8)	186 (60.2)	2.19 (1.64-2.91)	< 0.001
Winter	36 (17.2)	173 (82.8)	6.95 (4.67-10.34)	< 0.001
**Day of Month**				
0-10	255 (50.8)	247 (49.2)	-	-
11-20	290 (53.9)	248 (46.1)	0.88 (0.69-1.13)	0.316
21-30	337 (52.1)	310 (47.9)	0.95 (0.75-1.2)	0.664
**Time of Day**				
1-5	23 (21.7)	83 (78.3)	-	-
6-11	210 (48.2)	226 (51.8)	0.3 (0.18-0.49)	< 0.001
12-17	309 (56)	243 (44)	0.22 (0.13-0.36)	< 0.001
18-24	340 (57.3)	253 (42.7)	0.21 (0.13-0.34)	< 0.001

^a^Data are presented as No. (%).

^b^Abbreviations: OR, odds ratio; and CI, confidence interval.

## 5. Discussion

Regarding the data of those who were involved in road crashes, our analysis showed no significant differences between females and males in injuries and mortality rates; however, the mortality rate of females in road crashes with causalities was just merely higher than that of males. It seems that female are less skilled in driving in comparison to males in Iran and therefore, they would be involved in more serious crashes. This is the main reason to prioritize the development of a sex-oriented driving education program in Iran. Authors of some studies such as Spoerri et al. (14), Zhao et al. (5), Tiwari et al. (6), Vorko-Jovic et al. (7), and Valent et al. (9) explained a higher mortality rate in males than in females. Ravera et al. (15) reported that female drivers were involved in much more serious crashes than male drivers were because they more frequently drive under influence of drugs than male drivers do. We determined age as an important risk factor in road crashes in Iran. People aged between 25 and 34 years had higher odds of being injured during road crashes. People aged ≥ 54 years had higher mortality rate in road crashes. Previous studies in Iran stated that younger drivers had higher odds of being involved in road crashes (4, 16-18). Young drivers drive with higher speed and their poor experiences and driving skills makes them more prone to be involved in more serious road crashes (19). Lee et al. suggested double odds of mortality in aged people than young drivers (20). Massie et al. reported that the mortality rate was higher in old people than young people when they had been involved in road crashes (21). We have to speculate that young drivers were involved in much more serious road crashes and their road crashes had much more financial and psychosocial effect on their society. On the other hand, older people had poor visual acuity, weaker stature and skeleton, slow responses and reflexes, and less flexibility; hence, when they were involved in a road crashes, the outcome would be the worst. In our study, education level was a risk factor in road crashes but we could not find any direct association between this variable and crashes with causalities. In a study by Spoerri et al., a basic level of education was considered as higher odds to get involved in crashes with mortalities (14). In Iran, professional drivers who drive commercial vehicles have lower level of education but drivers with higher level of education drive usual/light vehicles, which are involved more frequently in road crashes; therefore, their mortality rate would be higher than driver with low level of education. In our study, we found that the time of road crashes occurrence had a direct association with the seriousness of the road crashes. Road crashes in winter and autumn were associated with higher rate of mortality but the odds of getting involved in road crashes with causalities in these seasons were lower than road crashes in summer. We have to conclude traffic density in roads in summer as a dependent variable should be considered as a main factor on the road crashes impact. In addition, we have to consider wintertime weather condition as a strong variable, which can intensify the road crashes impact. Several studies have evaluated the association between weather condition and impact of the road crashes. In one study by Valent et al., road crashes that had happened between January and March had a higher mortality rate (9, 22-25). We also found the same results in other studies concerning crashes that had been happened in winter (24, 25). One of the other variables that should be considered in road crashes impact is altered behavior of drivers in different days of a month. Based on our analysis, road crashes that had happened in the last ten days of the Persian months were more often associated with causalities; however, the same associations could not be found between drivers’ behavior and fatality rate. Some authors in previous studies have discussed the association between road crashes impact and the time of the day when the road crashes had happened (9, 24-26). All of these studies showed that there was a strong association between road crashes impact and the time of day and the season of the year when the road crashes had been occurred. Road crashes that had happened between 1 AM to 5 AM were strongly associated with higher mortality rate (5, 7, 9, 27) and had the worst impacts because within this period of time not only the road visibility is decreased but also divers are more tired and drowsy. 

Several studies have shown the association between road crashes impact and having the seatbelts on (5, 28-32). When they have their seat belts on, the impact of the road crashes will be less severe in case of human casualties and mortalities; however, it has no direct effect on the road crash incident at all. We have to consider the status of fastening seatbelts as an indirect cause for human causalities in road crashes but it has an inconsistent effect on human mortalities. We have to mention that the association between road crashes and fatalities is multi-factorial. In our study, when we considered age as a main risk factor in road crashes, fastening seatbelts showed different effect on the road crashes impacts, especially on mortality rate. Similar to the previous studies, the results of fastening seatbelt had no difference among different age group in our study. In the study by Rivara et al. (33), having seatbelts on, especially for children and elderly, had a preventive effect on human injuries and mortalities. 

Our study had its own points of strengths and limitations. One strength point of our study was the method of selecting samples as one of the strongest point of strength in this study. We randomly selected samples from all of the roads in Iran and therefore, we can generalize the results of our study to the whole country.

The limitations to our study included:

1. There was a lack of a definition for different types of injuries in our study.
2. Some of the road crashes with no causalities and low-cost damages were not registered in the country by involved drivers.
3. In some instances, police officers did not report the crashes completely.
4. There was not a defined purpose for our study in first place at all.
5. Under-reporting the crashes and injuries by police reports.

Despite some limitations, our study would be a valuable source of information regarding the effect of the main risk factors on road crashes in Iran.

According to our results, some variables are associated with high fatality rate in road crashes in Iran. These variables include age ≥ 55 years, level of education, female sex, winter season, and the time of the day when the crash happens, especially crashes that occur between 1 AM to 5 AM According to our results, all drivers in our country especially older drivers, female drivers, and drivers with higher education level had to be trained to decrease the intensity of the road crashes impacts. In addition, we need to improve the roads condition by installing proper equipment for providing enough visibility in darkness and preventing slipperiness during winter season, especially between 1 AM to 5 AM
